# The occlusion rates after distal radial access: how to multiply your bullets

**DOI:** 10.1186/s12916-024-03470-4

**Published:** 2024-06-13

**Authors:** Alexandru Achim, Zoltan Ruzsa

**Affiliations:** 1 Department of Cardiology, Niculae Stancioiu” Heart Institute, University of Medicine and Pharmacy “Iuliu Hatieganu”, Motilor 19-21, Cluj-Napoca-Napoca, 400001 Romania; 2 Internal Medicine Department, Division of Invasive Cardiology, University of Szeged, Szeged, Hungary

**Keywords:** Distal radial access, Radial artery occlusion, Ultrasound, Snuffbox

## Background

In this issue of *BMC Medicine*, Chen et al. published the results of the CONDITION trial (Comparison of Long-term Radial Artery OcclusiON in Coronary Diagnosis and/or Intervention Via DIstal vs. ConvenTIONal Transradial Access), a randomized controlled trial comparing the long-term radial artery occlusion (RAO) rates (at 3 months) with distal radial access (DRA) vs. conventional transradial access (TRA). The study showed a downstream benefit of the DRA, with lower occlusion rates and higher spontaneous recanalization rates—results of major clinical significance in the realm of repeated percutaneous interventions [1]. This commentary offers a concise analysis of the trial, aiming to elucidate the reasons behind conflicting results in prior research on RAO—an endpoint deemed controversial yet invaluable to operators.

### Main text

Transradial coronary catheterization, while offering numerous advantages such as reduced bleeding, improved hemostasis, and enhanced postprocedural nursing care, does pose the risk of various complications. These complications may include RAO, forearm hematoma, pseudoaneurysms, arteriovenous fistula formation, compartment syndrome, radial artery perforation, nerve damage, and local infections. Notably, RAO emerges as the most prevalent complication, with an incidence ranging from 1 to 30%, depending on the timing of assessment [2].

It is widely recognized that sheath-to-artery mismatch serves as a predictor for RAO. In other words, the larger the sheath or the smaller the artery, the higher the chances for an artery to be traumatized by catheters and to thrombose after extraction. In the current era of complex and repeated transcatheter interventions within the same patient, RAO emerges as the major “complication” or challenge of this approach (primarily posing a technical inconvenience for operators rather than patients, as RAO is mostly asymptomatic), essentially limiting radial access to a one-time opportunity.

The transradial approach, while offering significant advantages, also has the *Achilles’ heel* of RAO, which the femoral approach does not possess. To address this gap, DRA was received with great enthusiasm. In theory, the RAO rate associated with DRA is much lower, primarily due to the puncture being performed distal to the superficial palmar arch, which would perfuse the artery in case of trauma at the puncture level, preventing RAO [3, 4]. In practice, however, the randomized controlled DISCO RADIAL trial comparing proximal radial access vs. DRA did not demonstrate a significant difference, as long as hemostasis was conducted correctly—that is, with a “patent” hemostasis, not aggressive, leaving flow in the artery and a hemostasis band/dressing that was progressively decompressed from hour to hour [5]. While initially negative (and somewhat disappointing), this study prompted a necessary discussion on factors beyond patient anatomy, focusing on operator technique and post-care protocols. Addressing this, Chen et al. took the analysis to the next level, introducing radial ultrasound (US) examination as an indispensable step for DRA [6]. This was conducted by the same remarkable Chinese team in another large study of over 800 patients who underwent US examination both before and after the procedure, in search for predictors of RAO (distal RAO in their case). The findings unequivocally demonstrate that small arteries coupled with large catheters increase the risk of distal RAO (a DRA inner diameter/sheath outer diameter < 1 was an independent risk factor for occlusion) [6]. Systematic US should be then performed before the puncture to decide if the artery is technically suitable for intervention [7]. Additional factors potentially contributing to the disparate outcomes between the CONDITION and DISCO trials include a more uniform puncture approach in the Chinese study (conducted at a single center with five operators only), with a possibly more standardized hemostasis protocol (in the DISCO trial the hemostasis was managed “per hospital practice,” whereas in the CONDITION trial, an elastic bandage was used and hourly loosened), and an extended follow-up period for the RAO endpoint (3 months in the CONDITION trial vs. assessment up to discharge in the DISCO trial) [1, 5].

Chen et al. demonstrated that the value of DRA is seen in the long run, over the course of several months, being the first randomized controlled trial that evaluated the long-term incidence of RAO [1]. Their findings revealed that the incidence of DRA-associated RAO was significantly lower compared to proximal transradial access after a period of 3 months (0.8% vs 3.3%, *P* = 0.02), and that two thirds of DRA-RAO at 24 h recanalized spontaneously at 3 months, while only 1 half of the occluded arteries following conventional radial access re-opened at 3 months follow-up [1]. The trial demonstrated another important aspect in the pathophysiology of RAO. It is that acute thrombotic RAO can undergo spontaneous recanalization, occurring at a rate of around 50–60% within 30 days (50% in TRA vs 68% in DRA) [1]. Notably, the design of CONDITION did not include intraprocedural US, i.e., US-guided (not palpation-guided) puncture, but other studies did, demonstrating proven benefits [8]. The versatility of DRA expands to larger sheaths as well (7–8 French) where inevitably the chance of RAO increases significantly and where US plays the role of screening a sufficiently sized artery diameter, while DRA’s anatomical characteristics may further mitigate RAO risk [9]. The DISCO Radial trial [5], while not providing specific data on US usage, indicates that many recruiting centers routinely incorporated US during radial puncture. It should also not be forgotten that RAO can be recanalized, and here, both DRA and US are independently extremely important [10].

## Conclusion

In conclusion, it is evident that the reduction of RAO by DRA is not solely attributable to its inherent characteristics; the meticulous and careful practices of the operators are equally pivotal. DRA is not universally applicable, and US, with its dual function of preprocedural screening for artery diameter and periprocedural assistance in ensuring a clean puncture, serves to identify ideal candidates for DRA while excluding those who would not benefit from this elaborate technique. Thus, RAO becomes a controllable and preventable complication.

## Data Availability

No datasets were generated or analysed during the current study.

## Abbreviations

DRA: Distal radial access
RAO: Radial artery occlusion
TRA: Transradial access
US: Ultrasound
